# Identifying the Tuskegee Syphilis Study: implications of results from recall and recognition questions

**DOI:** 10.1186/1471-2458-9-468

**Published:** 2009-12-16

**Authors:** Ralph V Katz, Germain Jean-Charles, B Lee Green, Nancy R Kressin, Cristina Claudio, MinQi Wang, Stefanie L Russell, Jason Outlaw

**Affiliations:** 1 Department of Epidemiology & Health Promotion, NYU College of Dentistry, 250 Park Ave South, NY, NY 10012, USA; 2 Department of Oral Maxillofacial Pathology, SUNY at Buffalo School of Dental Medicine, 355 Squire Hall, 3435 Main St, Buffalo, NY 14214, USA; 3 Office of Institutional Diversity, H. Lee Moffitt Cancer Center & Research Institute, 12902 Magnolia Drive, Tampa, FL 33612, USA; 4 Healthcare Disparities Research Unit, Section of General Internal Medicine, Boston University School of Medicine and Department of Veterans Affairs Health Services Research & Development Service, VA Boston Healthcare System, 150 S. Huntington Ave, Bldg 9, 4th Fl, Boston, MA 02130, USA; 5 Department of Community Dentistry, University of Puerto Rico School of Dentistry, Medical Sciences Campus, PO Box 365067, San Juan, PR 00936-5067, Puerto Rico; 6 Department of Public and Community Health, University of Maryland School of Public Health, Suite 2387, Valley Drive, College Park, MD 20742, USA; 7 Harvard University School of Dental Medicine, 188 Longwood Ave, Boston, MA 02115, USA

## Abstract

**Background:**

This analysis assessed whether Blacks, Whites and Puerto-Rican (PR) Hispanics differed in their ability to identify the Tuskegee Syphilis Study (TSS) via open-ended questions following lead-in recognition and recall questions.

**Methods:**

The Tuskegee Legacy Project (TLP) Questionnaire was administered via a Random-Digit Dial (RDD) telephone survey to a stratified random sample of Black, White and PR Hispanic adults in three U.S. cities.

**Results:**

The TLP Questionnaire was administered to 1,162 adults (356 African-Americans, 313 PR Hispanics, and 493 non-Hispanic Whites) in San Juan, PR, Baltimore, MD and New York City, NY. Recall question data revealed: 1) that 89% or more of Blacks, Whites, and PR Hispanics were not able to name or definitely identify the Tuskegee Syphilis Study by giving study attributes; and, 2) that Blacks were the most likely to provide an open-ended answer that identified the Tuskegee Syphilis Study as compared to Whites and PR Hispanics (11.5% vs 6.3% vs 2.9%, respectively) (p ≤ 0.002). Even when probed by a recognition question, only a minority of each racial/ethnic group (37.1%, 26.9%, and 8.6%, for Blacks, Whites and PR Hispanics, respectively) was able to clearly identify the TSS (p < 0.001).

**Conclusions:**

The two major implications of these findings for health disparity researchers are 1) that it is unlikely that detailed knowledge of the Tuskegee Syphilis Study has any current widespread influence on the willingness of minorities to participate in biomedical research, and 2) that caution should be applied before assuming that what community leaders 'know and are aware of' is equally 'well known' within their community constituencies.

## Background

The current mandate for the NIH health disparities research agenda not only addresses the need for community-based epidemiologic studies that focus on the health disparities themselves, but also requires investigations that will provide a deeper understanding of the myriad factors that have contributed over the decades to the current disparities in health so evident across US subpopulations [1]. One of the leading health disparity agenda items is the need to achieve inclusion of African-Americans, as well as other minority groups, in biomedical research studies to ensure that study findings truly apply to all U.S. residents [2].

The U.S. Public Health Service (USPHS) Syphilis Study at Tuskegee, commonly referred to as the Tuskegee Syphilis Study (TSS), foisted research abuses on 399 African-American sharecroppers in Macon Country, Alabama who were the subjects in this 40 year study of the effects of untreated syphilis in the Negro male [3]. To this day the USPHS Syphilis Study at Tuskegee (1932-72) remains as the most infamous example of biomedical research subject abuse in U.S. history [4-31].

While some studies have reported on the broader related issue of distrust towards biomedical research in the minority communities, and generally reported a higher distrust within minority populations, [23-26] a few reports have pointed out the need to also recognize the widespread belief that an enduring 'legacy' of the Tuskegee Syphilis Study, namely that as a result of that study, African-Americans have a greater reluctance to participate in clinical research studies [7,27,28]. Two recent U.S. multi-city surveys using an in-depth survey questionnaire, investigated this 'legacy' of the TSS and found no evidence to support this widespread belief in either African-Americans or Whites [30,31].

Establishing the level of knowledge about the Tuskegee Syphilis Study, especially in Blacks, is a logical prerequisite to truly and accurately understanding its impact on minority research participation. One recent report using traditional close-ended questions (i.e., a 7 item true-false Facts- and-Myth quiz of detailed knowledge about the TSS) found that detailed knowledge about the facts of the Tuskegee Syphilis Study was uniformly low in both Blacks and Whites [32].

The goals of this report from our 3-City Tuskegee Legacy Project (TLP) Study were: 1) to describe the comparative level of identification of the Tuskegee Syphilis Study (TSS) in Blacks vs Whites vs Puerto Rican Hispanics separately for recognition type questions and for recall type questions; 2) to ascertain whether people held detailed knowledge of the TSS at the most accessible level of memory retrieval (i.e., triggered by a recognition probe) or at a less accessible level of memory retrieval (i.e, triggered by a recall probe); and 3) to discuss the implications of any observed differences for the recruitment of minorities into biomedical studies.

## Methods

### Overview

The 3-City TLP Study was designed to administer the Tuskegee Legacy Project (TLP) Questionnaire via random-digit dial telephone interviews to a stratified random sample of Blacks, non-Hispanic Whites and Puerto-Rican Hispanics aged 18 years and older in three cities: New York City, NY; Baltimore, MD; and San Juan, PR. The choice of these three cities was based upon obtaining the desired sample size for the three ethnic/racial groups within the broader parameters set by the goals of the projects within the NYU Oral Cancer RAAHP* Center (* = *Research on Adolescent and Adult Health Promotion*), a U54 Oral Health Disparities Research Center funded by the National Institute of Dental Craniofacial Research (NIDCR) at the National Institutes of Health (NIH). The data collection phase was conducted in the four month period of September-December 2003. This study was approved by the IRB of New York University.

The primary research instrument was the TLP Questionnaire, a 60 item instrument that addresses a range of issues related to the recruitment of minorities into biomedical studies. Details on the history and development of the TLP Questionnaire have been published elsewhere [5,19,20,30-32]. While the primary contrast for this TLP Study focused on the comparison of Blacks vs Whites on this historical issue in U.S. race relations, a second minority group (Puerto-Rican Hispanics) was included to determine whether the findings would they be generalizable to another minority groups. The TLP Questionnaire was administered in Spanish for all San Juan subjects, while Puerto-Rican Hispanics in New York City were given the choice of having the TLP Questionnaire administered either in English or Spanish.

**Figure 1:**
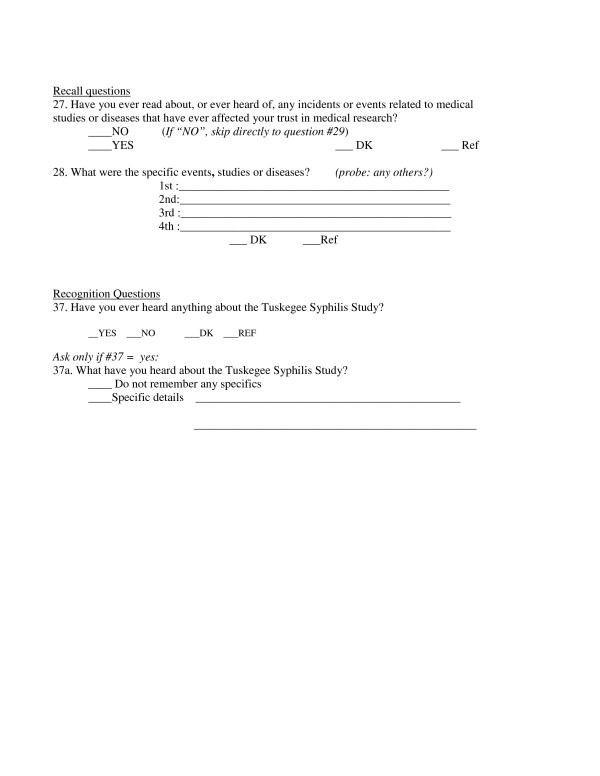
**The recall and recognition questions from the Tuskegee Legacy Project (TLP) in the 3-City TLP Study.**.

Figure 1 shows the precise wording of four questions from the TLP Questionnaire that comprised the open-ended recall and recognition probes which were the dependent variables in this study. The first two questions (Q27 and Q28) comprise the recall probe, in which the name 'Tuskegee Syphilis Study' is never mentioned, while the recognition probe (Q37 and q37a) directly asks about the TSS. All respondents who answered 'yes' to either of the lead-in questions (Q27 or Q37) were then asked the follow-up open-ended question, either Q28 "What were the specific events, studies or diseases?" for the recall question, or Q37b "What have you heard about the Tuskegee Syphilis Study?" for the recognition question. Each response to these open-ended questions was followed by the probe inquiry "Any others?", until the subject's responses were exhausted.

The variable of age was calculated from the 'date of birth' variable on the TLP Questionnaire, then classified into 5 age group categories for analyses. The level of education and level of income variables were collected in ordinal listings of nine ascending categories of educational level and of ten ascending categories of income level; each was then subsequently collapsed into five categories for the demographic table analyses. Chi squared analyses, a conservative approach, were performed for all statistical analyses in this report using SPSS v14. No software word recognition programs were utilized for analysis of any of the open-ended questions.

The schema developed for the analysis of the recall open-ended question (Q28) had four mutually exclusive, hierarchical categories based on degree of certainty that the subject had, in fact, identified the TSS: 1) Definitely Identified the TSS (i.e., used either the name Tuskegee or gave clear attributes); 2) Most Likely Identified the TSS (i.e., did not name the TSS but gave attributes likely linked to the TSS); 3) Questionable Identification of the TSS (i.e., did not name the TSS but gave 'could be' attributes that might link to the TSS); and, 4) Did Not Identify the TSS (i.e., neither named the TSS nor give any attribute that even possibly linked to the TSS). All open-ended responses were categorized by one evaluator into of the four above categories, followed by a series of debate and discussion review sessions with two other evaluators with the goal of achieving consensus placement of each open-ended response into one of the four categories.

The same 'debate and discussion' review process, with the same three evaluators, was used for the development of a two-tiered schema for the categorization of the open-ended recognition question (Q37a). All open-ended responses to the recognition question were initially put into one of 15 sub-categories and then those initial 15 sub-categories were subsequently classified into a final set of three categories: 1) Factually Correct TSS Details; 2) Myth (i.e., factually incorrect) TSS Details; and, No TSS Details.

The Factually Correct TSS Details category consisted of: 1) people used as guinea pigs; 2) study on African-Americans without their knowledge; 3) people left untreated; 4) study about African-Americans and syphilis; 5) an experiment; and 6) government lied. The Myth TSS Details consisted of sub-categories: 7) syphilis was given to people; 8) study done on soldiers; 9) wrong medication/treatment given; 10) university studies; 11) study done on blacks vs whites; 12) study done on prisoners; and, 13) illness caused by treatments. Finally, the third category of No TSS Details consisted of: 14) only facts about the disease of syphilis; and 15) responses totally irrelevant to the TSS.

### IRB Approval

This study was approved as 'Exempt from Review' by the NYU IRB.

## Results

In this study, the TLP Questionnaire was administered to 1,162 adults (356 African-Americans, 313 Puerto-Rican Hispanics, and 493 non-Hispanic Whites) in three cities: San Juan, PR, Baltimore, MD and New York City, NY with response rates by city, of 52%, 51% and 44%, respectively. The overall completion rate (# of completed interviews/# of initiated interviews) was 82.6%. Details on the age, sex, education, and income distribution of the 1,162 subjects within the three racial/ethnic groups in this 3-City TLP Study have been presented elsewhere [20,32].

Table 1 shows the percentage the respondents who answered 'yes' to the lead-in questions to the recall and recognition probes (i.e., Q27 & Q37, respectively), as well to the findings from a categorization of their responses to their respective follow-up open-ended responses. Column 1 in Table 1, which shows the percentage of 1,162 respondents who answered 'yes' for the recall probe (i.e., Q27) revealed no statistically significant differences by race/ethnicity, sex, or city but showed statistically significant upward trends with increasing education and income; the statistically significant differences across age groups revealed the youngest and oldest adults had lower percentages of 'yes' answers than the three middle age groups.

**Table 1:** 

		←------------RECALL probe------------→			←RECOGNITION probe→	
		Column 1 →	Column 2 →	Column 3	Column 4 →	Column 5
		% 'yes' to recall probe (Q27)	% of 'yes' on Q27 who 'Definitely' or 'Most Likely'	% of 'yes' on Q27 who 'Definitely' identified	% 'yes' to recognition probe (Q37)	% of 'yes' on Q37 who 'clearly' identified
Variable	Strata	(n = 1,162)	identified the TSS	the TSS	(n = 1,162)	the TSS*
race/ethnicity^2,3,4,5^	Blacks	54.5	20.7	11.2	56.2	66.0
	Whites	55.3	9.0	6.3	38.5	70.0
	Puerto Rican Hispanics	51.4	5.7	4.3	24.3	35.5
						
sex^2,3,4^	females	52.9	9.9	5.8	37.6	60.4
	males	56.2	15.5	10.2	44.9	66.3
						
city^3,4,5^	Baltimore, MD	56.3	13.9	7.9	48.8	71.2
	New York City, NY	53.8	13.6	8.6	40.9	62.7
	San Juan, PR	50.8	1.3	1.3	21.8	28.2
						
age (yrs)^1,4^	18-29	45.7	8.5	8.5	28.1	55.8
	30-44	55.3	15.4	9.1	42.5	61.9
	45-59	60.9	11.7	7.4	46.6	67.5
	60-74	55.9	7.7	2.6	38.5	62.3
	74+	33.7	10.0	5.0	32.5	51.9
						
education^1,2,3,4,5^	< H.S. grad	35.3	0.0	0.0	25.8	38.8
	H.S. grad	49.8	8.3	4.6	27.2	52.8
	some college	56.7	16.7	11.1	43.3	64.6
	college grad	59.7	10.6	6.4	50.3	65.8
	postgrad/prof degree	72.5	17.2	10.1	56.4	77.4
						
income^1,2,3,4^	< $20,000	42.9	3.5	3.5	26.1	45.2
	$20,000 - 34,999	55.6	5.4	1.4	35.8	56.9
	$35,000 - 49,999	55.1	16.5	7.5	46.7	65.1
	$50,000 - 74,999	67.9	19.7	18.2	51.5	72.5
	$74,999+	73.9	17.2	10.1	58.8	75.6

* *'clearly identified the TSS' included subjects who gave either a Factually Correct TSS Detail or a Myth TSS Detail*

^1 ^= statistically significant for 'yes' to recall probe (χ^2^, p < 0.001) for frequency distribution on this variable within Column 1

^2 ^= statistically significant for % 'yes' to recall probe who named the TSS (χ^2^, p < 0.05) for frequency distribution on this variable within Column 2

^3 ^= statistically significant for % 'yes' to recall probe who named or most likely identified the TSS (χ^2^, p ≤ 0.05) for frequency distribution on this variable within Column 3

^4 ^= statistically significant for recognition probe (χ^2^, p < 0.05) for frequency distribution on this variable within Column 4

^5 ^= statistically significant for % 'yes' to recognition probe who most likely identified the TSS (χ^2^, p < 0.05) for frequency distribution on this variable within Column 5

Of the 532 subjects who answered 'yes' to the lead-in for the recall question (Q27) and then gave a valid response to the open-ended follow-up question, Column 2 in Table 1 shows the percentage of respondents who either 'Definitely or Most Likely' identified the TSS in their open-ended answer and revealed statistically significant differences by race, sex, education and income with Blacks and males as well as respondents with higher education and higher income all being more likely to have directly named Tuskegee. Similarly, again among these same 532 respondents, Column 3 shows the subset from Column 2 who 'Definitely' identified the TSS and largely echoes the trends in statistically significant findings as seen in Column 2.

Finally, Columns 4 and 5 in Table 1 show the parallel findings for the recognition probe lead-in question (Q37). Column 4 shows the percentage of the 1,162 respondents who answered 'yes' (n = 466), and revealed statistically significant differences across strata for each of the demographic variables. Blacks, males, residents of Baltimore, and 45-59 year olds were the most likely to say 'yes' within those demographic variables, and both education and income, as with the recall probe, showed statistically significant upward trends with increasing education and income, with the percentage saying 'yes' more than doubling from the lowest to the highest category. The fifth, and last, column in Table 1 presents, for the 40.1% (n = 466) who answered 'yes' to the lead-in probe for the recognition question (Q37), the percentage who 'clearly' identified the TSS by naming relevant study attributes. In this column, statistically significant differences were observed across racial/ethnic groups (with Puerto Rican Hispanics at 35.5% at about one-half the rates for Blacks and Whites) and across cities (with San Juan at 28.2% at less than one-half the rates for Baltimore or New York City), as well as for education and income both of which showed a positive correlation with a doubling from lowest category to highest for education, the stronger relationship of the two.

**Figure 2:**
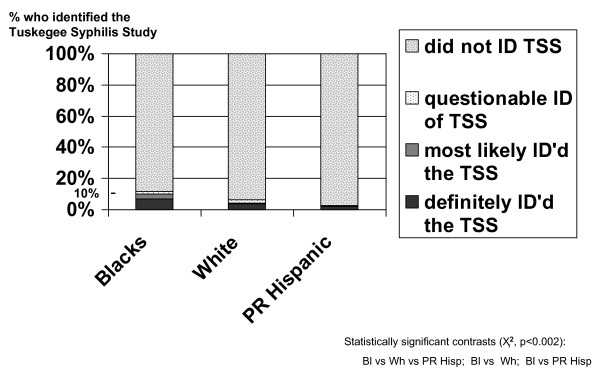
**The open-ended recall question: percentage of all 1,162 respondents by race/ethnicity who identified the Tuskegee Syphilis Study in the 3-City TLP Study**.

Fig 2 shows the distribution of their open-ended replies to the recall question (Q28) categorized into one of the four response categories. These data revealed: 1) that 90% or more of Blacks (90.2%), Whites (95.9%) and Puerto Rican Hispanics (97.3%) were not able to name or identify the TSS; and despite this, 2) that Blacks were the most likely to provide an open-ended answer that at some level identified the TSS, as compared to Whites and Puerto Rican Hispanics (11.5% vs 6.3% vs 2.9%) with the two way contrasts between Blacks vs Whites and between Blacks vs Puerto Rican Hispanics also being statistically significant (all p values < 0.002).

**Figure 3:**
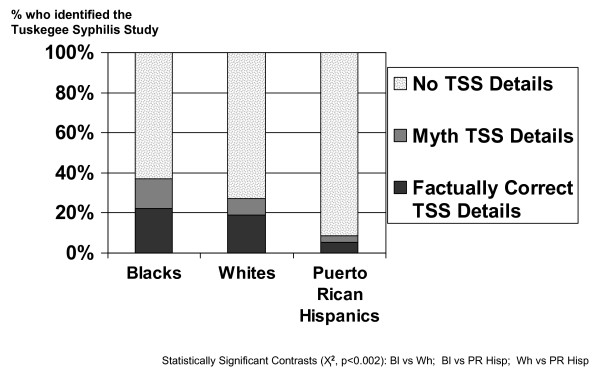
**The open-ended recognition question: percentage of all 1,162 respondents by race/ethnicity who identified the Tuskegee Syphilis Study in the 3-City TLP Study**.

Fig 3, which shows the comparable data for the open-ended recognition question (Q37a), reveals that: 1) only a minority of each racial/ethnic group was able to clearly identify the TSS (37.1% of Blacks, 27.2% of Whites, and 8.6% of Puerto Rican Hispanics provided either Factually Correct TSS Details or Myth TSS Details, p < 0.001); and, 2) of the three racial/ethnic groups, Blacks were most likely to 'clearly identified the TSS' either 'factually' (22.2% gave Factually Correct TSS Details) or as 'a myth' (14.9% gave Myth TSS Details) as compared to Whites (18.9% and 8.3%) or Puerto Rican Hispanics (5.4% and 3.2%) (p < 0.001).

## Discussion

These findings indicate, not surprisingly, that more individuals hold a 'vague impression' of having heard of a negative medical event which would affect their trust in research than can 'name a specific event'. More poignantly for this analysis, only 11.8% of all 1,162 subjects were able to identify the TSS by either by name (Definitely) or attribute (Most Likely) in response to the recall probe (with only 7.3% actually naming stating Tuskegee). These data clearly show then that most people do not have recall memory about, much less routinely think about, the TSS when asked about incidents of medical research abuses. That being true for the sample as a whole, these data also show that 20% of Blacks who said they did recall such a medical incident were able to identify the TSS, either by name or by attributes, and that this was twice as common as among Whites. These data also clearly show that for Puerto Rican Hispanics, especially those living in San Juan, recall of the TSS is a relatively rare event.

While there was a direct positive correlation on the recall probe between education and income levels with the ability to identify the TSS, the highest rates for subgroups within any of the education and income strata did not equal the overall rate among Blacks. Clearly, these data attest to the continuing relative importance of the TSS within the Black community.

The comparable findings from the open-ended recognition probe, as also shown in Table 1, reinforce the direction of the findings from the recall probe as regards the demographic variables, i.e., Blacks and Whites were approximately twice as likely as Puerto Rican Hispanics to clearly identify the TSS by citing attributes. Further, while these same patterns were seen for the recognition probe across increasing levels of education and income, as had been observed above for the recall probe, this effect was more muted in response to the recognition probe.

These findings of low detailed knowledge via open-ended recall and recognition probes about the TSS from our 3-City TLP Study are in keeping with our previous findings from a set of seven close-ended questions that comprised the TSS Facts and Myth Quiz, an element within original TLP Questionnaire as administered in 1999, i.e., mean number of correct responses (± s.d.) was essentially the same for Blacks (1.6 ± 1.4) and for Whites (1.7 ± 1.3). with over 90% of the respondents who had heard of the TSS having a Facts and Myth Quiz score of 3 or less [32]. Further, our findings on low detailed knowledge of the Tuskegee Syphilis Study are in keeping with the findings other investigators [17,18].

One major implication of our findings for researchers conducting health disparity community-based studies is the general caution against assuming that the general population 'knows' what community leaders may 'know and operate on' as everyday awarenesses and detailed knowledge. Clearly, from the written articles about the TSS in both the lay and professional literature for the first 20 years following the termination of the TSS based upon intense newspaper coverage in 1972, community leaders were well aware of the TSS and its projected (albeit assumed vs scientifically evidence-based) related negative effect on the recruitment of minorities into biomedical studies [6,8,10,23-25,27-29]. To this day, many community leaders remain with these beliefs despite the more recent unfolding literature showing that this long-held belief in the 'legacy' of the Tuskegee Syphilis Study (i.e., Blacks were less willing to participate in biomedical studies because of their awareness and/or detailed knowledge of the TSS) is simply not supported by the studies that have looked directly into this issue, primarily over the past decade [10-22,26,30-34].

The second major implication of our study for researchers who conduct health disparities research, either in the community or in clinical settings, are based upon the specific findings in our study which clearly suggest that awareness of, or detailed knowledge of, the TSS is unlikely to have even a moderate impact on recruitment among Blacks (in particular) or other minorities (in general) into their biomedical studies, given the extremely low level of both awareness and/or detailed knowledge of the TSS in their communities, as revealed in our data. Further, logic suggests that as 'other reasons' must therefore account for any recruitment problems with potential minority subjects, that the investigators review, improve and intensify their 'on the ground' recruitment approaches, since any recruitment problems encountered are clearly not due to 'this past historical fact of the TSS' which would be far more nebulous to 'deal with' and to overcome. In point of fact, recent reports have documented that Blacks and Hispanics do enroll at equal yield rates to Whites when active, thoughtful, targeted recruitment plans are employed [33,34].

The third major implication of our findings lies within our methodological comparison of using recognition vs recall questions to probe for awareness and/or detailed knowledge of the TSS. Our findings fully support prior work that has found evidence supporting the theory that of the two types of information retrieval questions, recognition questions are of lesser complexity and more likely to trigger memory as the information is provided as a clue within the question itself, whereas in recall questions the subject must, devoid of clues, independently retrieve 'the memory' [35].

The two major limitations of this study both relate to 'inherent external phenomena': one of which puts limitations on the scope of our basic research question, while the other affects the degree of confidence one has in 'generalizing the findings' of this study. The first of these limitations is the fact that these data were collected in 2003, three decades after the termination of the TSS in 1972. Thus the research questions addressed in this report have the implicit caveat "as determined three decades after the termination of the TSS". Our findings do not provide direct evidence about, nor insight into these research questions as they might have been answered at post-study periods of 5, 10, or 20 years, and the answers to these research questions from those earlier time periods will never be known as there were no detailed studies prior to our Tuskegee Legacy Project.

The second major limitation of this study results from the 'ever-shrinking' response rates over recent years within the survey technique of Random Digit Dial (RDD) telephone surveys. As recently as the early 1990's, professional RDD survey firms would routinely achieve an 85% response rate as the 'standard of the day'. With the advent of caller ID and then rapid growth of cell phones, this once 'proud survey method' now has 'achievable response rate standards' set at 50-55%, which inherently limits the degree of confidence that one has in generalizing the findings to the communities surveyed. If this erosion in response rates continues, the day will come where the achievable response rates will be deemed as being 'just too low' for the technique to be considered a rigorous approach to gathering opinions of a given community which can be generalized to the reference population.

## Conclusions

This analysis of our TLP Study is the first report in the literature to quantify both the level of awareness of, and detailed knowledge about, the Tuskegee Syphilis (TSS) Study comparatively in Blacks, Whites and Puerto-Rican Hispanics. Further, it is also the first report in the literature to explore the methodological differences between using recognition vs recall questions to ascertain awareness and detailed knowledge of the TSS across racial/ethnic groups.

Overall, the TLP Questionnaire was designed to allow identification of factors related to recruitment of minorities into biomedical studies. This analysis used a segment of the TLP Q which focused on assessing respondents' level of accessible memory regarding detailed knowledge of the TSS provides unique findings which contribute to our fuller understanding of the role played by one major infamous historical biomedical research event (i.e., the TSS) in the 'biomedical recruitment scene' within the broader, ongoing 'life story' of U.S. racial relations. Thus, when the findings from this first in-depth study of identifying the TSS by detailed knowledge are combined with related prior studies on the TSS and distrust issues in minority recruitment, it appears that more individuals hold a vague impression of having heard of this negative medical event which would affect their trust in research, than can actually name or give details about that specific event.

## Competing interests

The authors declare that they have no competing interests.

## Authors' contributions

RVK: conceived and directed the study, led the development of the TLP Questionnaire and the data analysis, wrote the initial draft of the manuscript, and led the team's crafting of the final manuscript. GJC: led the development, analysis, and interpretation of the categorization schema for the open-ended questions, assisted by RVK and JO. BLG and NRK: were central to the development of the TLP Questionnaire, assisted with the writing of the grants that supported this research, helped plan the data analysis and data interpretation, and contributed to the writing of the final manuscript. CC: helped plan the data analysis and data interpretation, and contributed to the writing of the final manuscript and the development of the TLP Questionnaire. MQW and SLR: conducted the statistical analyses, help to plan and finalize the data interpretation and contributed to the writing of the final manuscript. JO: active participant in the development, analysis, and interpretation of the categorization schema for the open-ended questions. All authors read and approved the final manuscript.

## Pre-publication history

The pre-publication history for this paper can be accessed here:

http://www.biomedcentral.com/1471-2458/9/468/prepub
